# Risk Factors Indicating Difficulty During Gastric Endoscopic Submucosal Dissection for Inexperienced Endoscopists: A Retrospective Study

**DOI:** 10.7759/cureus.32713

**Published:** 2022-12-19

**Authors:** Kensuke Higuchi, Atsushi Katagiri, Shinya Nakatani, Kazuo Kikuchi, Takahisa Fujiwara, Toshihiko Gocho, Kazuya Inoki, Kenichi Konda, Fuyuhiko Yamamura, Hitoshi Yoshida

**Affiliations:** 1 Department of Medicine, Division of Gastroenterology, Showa University School of Medicine, Tokyo, JPN

**Keywords:** training, difficulty, beginner, endoscopic submucosal dissection, gastric neoplasia

## Abstract

Aim: Factors that may make endoscopic submucosal dissection (ESD) difficult for operators have been evaluated according to results based on the performance of experienced endoscopists. This study aimed to verify the predictors of difficult gastric ESD for ESD beginners.

Methods: From January 2015 to December 2021, 466 superficial gastric neoplasms were treated with ESD at Showa University Hospital. Excluding 103 lesions that performed ESD by experts who experienced more than 80 ESDs, a total of 363 lesions were included. The lesions were divided into two groups according to the ESD performance experience of the operator: ESD beginner (EB; ESD experience≤30 cases) and ESD intermediate (EI; ESD experience 31-80 cases) groups. Relationships between difficult ESD (having at least one of the following: procedure time>60 min, incomplete resection, change of operator, and occurrence of severe complications) and clinicopathological findings of the lesion were analyzed.

Results: The complete resection rates and the difficult ESD rates in the EB and EI groups were 99.3%, 94.8%, and 61.2%, 50.7%, respectively. In the EB group, univariate analysis showed that difficult ESD rate was significantly higher in the non-lower third lesions, the lesser curvature lesions, and cancerous lesions. In the EI group, univariate analysis showed that difficult ESD rate was significantly higher in lesion with ≥20 mm size, lesser curvature lesions, lesions with ulcers, and submucosal cancers. Multivariate analysis showed that the lesser curvature location and cancerous histology in the EB group and ≥20 mm lesion size, the lesser curvature location and submucosal invasion in the EI group were independent predictors of difficult ESD.

Conclusions: The lesser curvature location is recognized as independent ESD difficulty factor for both beginners and intermediates. Cases with lesions located in the lesser curvature should not be selected for gastric ESD training by beginners.

## Introduction

Endoscopic submucosal dissection (ESD) has been performed as a minimally invasive and effective treatment for early gastric cancer and dysplasia worldwide [1-3]. Compared to endoscopic mucosal resection (EMR), ESD has a higher en bloc resection rate and a lower risk of recurrence for gastric neoplasia ≥20 mm in size [4]. However, gastric ESD is more technically difficult to perform than EMR, and the resulting risk of gastric perforation is about 4-5% [5,6]. Due to its high technical difficulty, it has been reported that the self-completion rate of ESD by inexperienced endoscopists under supervision of experts is only 60% [7]. Several reports have examined the number of ESD operator experiences required to acquire basic ESD skills for a beginner. Oda et al. determined that 30 cases were necessary to acquire the basic technical skills for ESD, especially in the lower third of the stomach [8], and Gotoda et al. found that at least 30 cases of ESD operator experience were required for a beginner to gain early ESD proficiency [9]. Furthermore, Yamamoto et al. showed that the minimal amount of training to achieve preceptorship in ESD is performing at least 80 procedures [10].

Ex vivo models using animal organs and in vivo training on living animals have been reported to be used as training methods for ESD procedures [11,12]. The trainee can effectively learn basic ESD techniques using these models, such as marking, local injection, mucosal incision, and submucosal dissection. However, these training models require various preparations, such as equipment and facilities, which may hinder frequent ESD training. In Japan, gastric ESD for simple cases under the supervision of experts has traditionally been practiced as training for beginners [7,13]. Gastric ESD for humans differs from model-based ESD in terms of the responsibility required for ensuring the safety of the patient; therefore, it is believed that gastric ESD training on actual patients, with easier cases, should be introduced for beginners who have sufficiently practiced ESD on a model.

The difficulty in gastric ESD has been reported to vary depending on the lesion location and nature of the lesion. Lesions in the upper third of the stomach, large-sized lesions, cancerous lesions, and cases with submucosal fibrosis are considered to pose greater difficulties during ESD [14-16]. Imagawa et al. and Ahn et al. reported that the upper third location of lesion and larger lesion size were predictive factors for difficult ESD [14,15]. Kim et al. described that difficult ESD procedures are associated with larger lesion size and upper third location, submucosal fibrosis, and submucosal invasive carcinoma [16]. However, they did not consider the location of lesion in the cross-sectional circumference. Yoon et al. focused on the location of lesion in the cross-sectional circumference and described that posterior wall lesions and upper-third lesions were associated with incomplete resection and perforation in gastric ESD performed by attending gastroenterologists [17]. According to these reports, it is recommended that beginners in gastrointestinal endoscopy should perform ESD in cases without these difficult conditions. Oda et al. recommended that trainees should start performing ESD on lesions easier to treat including those that are located in the lower third of the stomach, smaller in size, and without ulcers [8]. However, it has not been investigated which location of the cross-sectional circumference is difficult for performing ESD by beginners.

In the present study, we conducted a retrospective analysis to verify the predictors of difficult gastric ESD including lesion location in the cross-sectional circumference, specific to inexperienced endoscopists.

## Materials and methods

Patients

**Figure 1:**
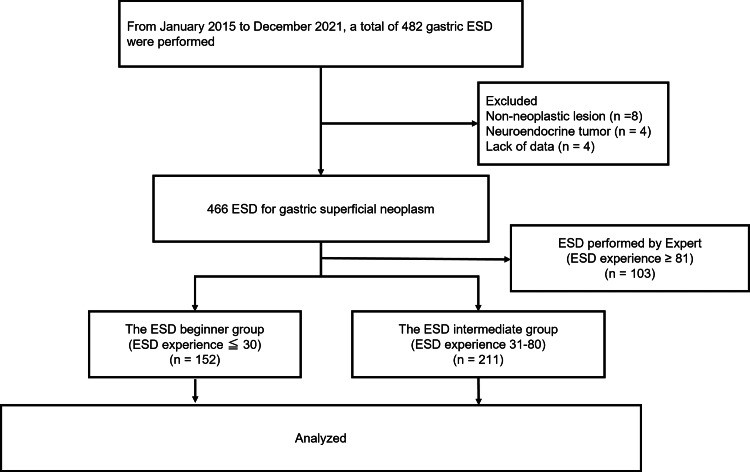
Study flow diagram showing number of gastric ESD performed between January 2015 and December 2021.

This retrospective single-center study included consecutive patients who underwent gastric ESD between January 2015 and December 2021 at the Showa University Hospital. During the study period, 482 gastric ESD procedures were performed. Of these, 12 procedures were excluded because the lesions were histologically diagnosed as non-neoplastic or neuroendocrine tumors. Four were excluded owing to missing data. A total of 103 cases were excluded due to ESD performed by experts. Finally, a total of 363 ESD procedures for superficial gastric neoplasms were analyzed (Figure 1).

Procedure

Patients on antithrombotic drugs were instructed to withdraw from the drug before the procedure for a specified period according to the guidelines for gastrointestinal endoscopy for patients taking antithrombotic drugs [18]. Administration of antithrombotic drugs was resumed the day after the procedure when no signs of bleeding were confirmed [19]. For patients at high risk of thromboembolism, ESD was performed with continued antithrombotic administration.

All ESD procedures were performed using a GIF-H290T or GIF-Q260J (Olympus Medical Systems, Tokyo, Japan). A disposable transparent hood (F-050; TOP Corp., Tokyo, Japan) was attached to the tip of the endoscope during ESD, in all cases. The endo knife was selected by the examiner, and either a Dual J-knife (KD-655; Olympus Medical Systems, Tokyo, Japan) or an insulated tip (IT-2, KD-611L; Olympus Medical Systems, Tokyo, Japan) was used. Both endo knives were allowed to be used in difficult cases such as lesions with fibrosis. Hemostatic forceps (Coaglasper, FD-410LR; Olympus Medical Systems, Tokyo, Japan) were used when arterial bleeding occurred during the procedure. High-frequency surgical equipment (VIO300D; Erbe Elektromedizin GmbH, Tübingen, Germany) was used for marking, incision of the gastric mucosa, dissection of the gastric submucosal layer, and hemostasis.

A 20% concentration of glycerin-fructose (Glyceol; Chugai Pharmaceutical, Tokyo, Japan), 2% epinephrine (Bosmin; Daiichi Pharmaceutical, Tokyo, Japan), and indigo-carmine (Alfresa-Pharma, Osaka, Japan) were used for submucosal injection; sodium hyaluronate solution (MucoUp, Boston Scientific, Massachusetts, USA) was also used in difficult cases.

The steps followed during the ESD procedure

To mark around the lesion, an endoscope with a magnifying function (GIF-H290Z; Olympus Medical Systems, Tokyo, Japan) was inserted into the stomach, and the extent of the lesion was confirmed using narrow-band imaging with magnification and chromoendoscopy. The normal mucosa around the lesion was marked with a small dot using a needle knife or dual knife 5 mm away from the edge of the lesion.

The endoscope used for the marking was removed and replaced with a treatment-dedicated endoscope (GIF-290TI or GIF-Q260J), with a transparent hood attached to the tip. A glycerol solution containing epinephrine and indigo carmine was injected into the submucosal layer using an injection needle immediately outside the marking to provide space for excision. The procedure was performed in a prograde or retrograde view depending on the lesion location.

A mucosal pre-cut hole was made on the normal mucosal side of the dot marking to insert the tip of the IT-2 knife. The holes were made on both sides (right and left sides in an endoscopic view) of the lesion, and the tip of the IT-2 knife was inserted into the submucosal layer through these holes. A continuous mucosal incision was made outside the marking dots to create a semicircular mucosal incision line proximal to the endoscopic tip. After submucosal injection, a mucosal incision line similar to that using IT-2 was created using the Dual-J knife.

Submucosal Dissection and Additional Mucosal Incision

The submucosal layer under the incised mucosa was dissected immediately after the first semicircular mucosal incision. After appropriate dissection, the submucosal layer under the lesion became clearly visible and was continuously dissected. Subsequently, an additional mucosal incision and dissection of the submucosal layer under the lesion were performed. If the submucosal bulge was insufficient, submucosal injections were repeatedly administered at any stage. Eventually, the mucosal incision became circumferential and all the submucosal layers beneath the lesion were dissected, resulting in en bloc resection of the lesion. The Endo Cut mode was used for mucosal incision and submucosal dissection. When a blood vessel was found in the submucosal layer to be resected, the submucosa was dissected using forced coagulation modes to prevent bleeding.

Hemostasis and Retrieval of the Resected Specimen

When hemorrhage from small vessels occurred, the bleeding point was coagulated with the blade of the IT-2 knife or tip of the Dual-J knife, using the forced coagulation mode. If the bleeding originated from a large blood vessel, or hemostasis could not be obtained after several coagulation using an endo knife, hemostasis was performed in the soft coagulation mode using hemostatic forceps (Coagrasper, Olympus Medical Systems, Tokyo, Japan). The resected specimen was gripped at the tip of the endoscope by continuous suction through the scope channel and retrieved from the patient's body by endoscope withdrawal.

Prevention of Delayed Bleeding

After retrieval of the specimen, the mucosal defect was washed with a water jet to remove adhered blood clots and exudates. The inner edge of the mucosal incision was coagulated using hemostatic forceps, and all the exposed blood vessels present in the mucosal defect were treated similarly.

Histopathological Diagnosis

The resected specimen was immediately stretched onto a thin urethane plate, affixed to a needle, and fixed with formalin. Experienced pathologists evaluated the completion of treatment and curability of the procedure. In all patients, oral vonoprazan fumarate 10 mg/day was administered from one week before the procedure to the day before the procedure. When permission to drink water was given, the day after ESD, vonoprazan fumarate 20 mg/day was administered orally and continued for eight weeks.

Sedation

During the procedure, sedation and analgesia were performed via intravenous injection of midazolam (Sandoz K.K., Tokyo, Japan) and pethidine hydrochloride (Takeda Pharmaceutical Company, Tokyo, Japan). When the patient complained of pain, additional doses were administered, as appropriate.

Operator

The beginner was defined as ESD operator with less than 30 ESD operator experience according to previous studies [9,20]. This study included six beginners with no experience in ESD training in both in vivo and ex vivo models. Those with 31-80 ESD operator experiences were treated as intermediate.

Policy to Change an Examiner to an Experienced Endoscopist

Beginners were allowed to be ESD operators if they met all the following conditions: (1) performed >500 cases of upper gastrointestinal endoscopy and 100 cases of colonoscopy; (2) mastered target biopsy, endoscopic hemostasis, and colonic endoscopic mucosal resection; and (3) assisted >20 cases of ESD procedures performed by experts to learn basic ESD maneuvers. All ESD procedures by beginners were performed under the supervision of one of three experienced endoscopists (AK, KK, and KI).

Beginners were replaced by a senior experienced examiner under the following conditions: (1) when hemostasis was not achieved by multiple hemostases by a beginner, or when the supervisor determined that it was challenging to continue ESD with a beginner due to technical difficulties; (2) when gastric perforation occurred; and (3) when the patient's physical activity was intense despite the administration of sedatives and analgesics, or when the patient's vital signs (e.g., blood pressure, saturation, and heart rate) were unstable and the supervisor decided that the procedure needed to be completed earlier.

The present study was conducted in accordance with the guidelines of the Declaration of Helsinki and was approved by the Showa University Review Board (No. 2775). The need for informed consent was waived by the institutional review board because the analysis used anonymous clinical data obtained after each patient agreed to treatment with written informed consent.

Definitions and parameters

All ESD procedures were divided into two groups: ESD performed by beginners (EB group) and intermediates (EI group). ESD procedures that met any of the following four criteria were defined as difficult ESD by modifying the definition of Kim et al. - (1) procedure time >60 min; (2) incomplete resection including piecemeal resection; (3) not a self-completion; and (4) any severe complication, such as gastric perforation, delayed bleeding, and aspiration [16].

Procedure time was measured from the start of mucosal marking until the end of tumor removal, including the time required to achieve hemostasis for intraoperative bleeding. Complete resection was defined as en bloc resection with pathologically tumor-free lateral and vertical margins. Self-completion was defined as an ESD procedure that was accomplished without replacing the operator with a supervisor. Delayed bleeding was defined as bleeding following ESD at the tissue defect diagnosed after 24 hours that required hemostasis. Aspiration associated with ESD was defined as low oxygen saturation (<93%) during or after ESD with radiographic imaging revealing pneumonia.

The macroscopic type of lesion was determined according to the Paris classification and divided into following three categories: protruded, flat, and depressed [21]. The presence of endoscopic ulceration was determined when scars with fold concentration coexisted in the lesion area.

**Figure 2:**
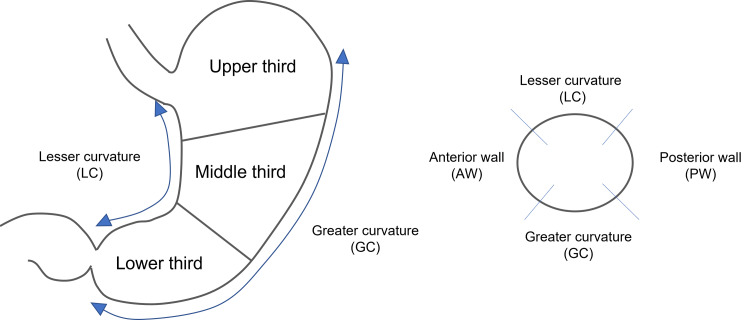
The location of the lesion was classified into the upper, middle, and lower thirds. The location in the cross-sectional circumference of the lesion was also classified as the anterior wall, posterior wall, lesser curvature, and greater curvature.

The location of the lesion was classified into the upper, middle, and lower thirds. The location in the cross-sectional circumference of the lesion was also classified as the anterior wall, posterior wall (PW), lesser curvature (LC), and greater curvature (GC). For lesions that occupied multiple locations, the central part of the lesion was considered to represent the location of the lesion (Figure 2).

Statistical analysis

A chi-squared test or Fisher’s exact test was used for categorical variables, and the Mann-Whitney U test was used for continuous variables in the univariate analysis. For three or more categorical variables, such as the location in the cross-sectional circumference, the Bonferroni method, was used for multiple comparisons. Multivariate logistic regression analysis was performed to analyze predictors with significant differences or correlations determined using univariate analysis. A p-value of <0.05 in each analysis was considered statistically significant. Statistical analysis was performed using the JMP statistical analysis software version 16.0 (SAS Institute, Cary, NC) for Windows.

## Results

Baseline clinicopathologic characteristics of the lesion and the clinical outcomes of ESD

The clinicopathologic characteristics of the 328 patients with 363 gastric neoplasia included in the present study are shown in Table 1. The mean tumor size was 20.7±14.0 mm. Most of the lesions were depressed (49.0%) and flat (45.7%). The lower third of the stomach (52.6%) and the LC in the cross-sectional circumference (44.3%) were the most common locations of the lesion. The lesions included 305 adenocarcinomas (84.0%) and 58 adenomas (16.0%). The proportion of submucosally invading cancers was 11.6%.

**Table 1:** Baseline characteristics of gastric neoplasia.

Characteristic	Total (n=363)
Male sex, n (%)	254 (70.0)
Age (years) mean±SD	75.3±8.5
Tumor size (mm) mean±SD	20.7±14.0
<20 mm, n (%)	199 (54.8)
≥20 mm, n (%)	164 (45.2)
Morphology, n (%)	
Protruded	13 (3.6)
Flat	166 (45.7)
Depressed	178 (49.0)
Mixed	6 (1.6)
Location, n (%)	
Upper third	50 (13.8)
Middle third	122 (33.6)
Lower third	191 (52.6)
Location in the cross-sectional circumference, n (%)	
Anterior wall	53 (14.6)
Posterior wall	71 (19.5)
Lesser curvature	161 (44.3)
Greater curvature	78 (21.5)
Ulceration (endoscopic), n (%)	9 (2.5)
Invasion depth, n (%)	
Mucosa	321 (88.4)
Submucosa	42 (11.6)
Ulceration (histologic), n (%)	12 (3.3)
Histology, n (%)	
Adenoma	58 (16.0)
Adenocarcinoma	305 (84.0)
Procedure time (min.) mean±SD	78.0 ± 51.0
<60 min., n (%)	170 (46.8)
≥60 min., n (%)	193 (53.2)
Self-completion, n (%)	333 (91.7)
Complete resection, n (%)	351 (96.7)
En bloc resection, n (%)	363 (100)
Severe complication, n (%)	21 (4.5)
Intra-procedual perforation	5 (1.1)
Delayed perforation	1 (0.2)
Delayed bleeding	12 (2.6)
Aspiration	3 (1.1)
Examiner, n (%)	
Beginner (1-30)	152 (41.9)
Intermediate (31-80)	211 (58.1)

The mean procedure time was 78.0±51.0 min. The complete resection rate was 96.7% and the en bloc resection rate was 100%. The delayed bleeding rates were 2.6%. Beginners performed gastric ESD in 152 patients (41.9%). Comparison of the clinicopathological findings of the lesion and clinical outcomes of ESD between beginner and intermediate groups are summarized in Table 2. Tumors located in the lower third region of the stomach were significantly more frequent in the EB group than in the EI group (66.4% vs 42.6%, p<0.001). Lesions with submucosal invasion (15.2% vs. 6.6%) were significantly more frequent in the EI group than in the EB group (p=0.017). Self-completion rate was significantly higher in the EI group than in the EB group (94.8% vs 87.5%, p=0.001). Complete resection rate was significantly higher in the EB group than in the EI group (99.3% vs 94.8%, p=0.017). There were no significant differences in lesion size, location in the cross-sectional circumference, malignant histology, pathological ulcer, procedure time, en bloc resection rate, and severe complication rates between the two groups (Table 2).

**Table 2:** Comparison of lesion characteristics between the ESD beginner group and the ESD intermediate group. *Statistically significant. ESD: endoscopic submucosal dissection

Characteristics	Beginner group (n=152)	Intermediate group (n=211)	p-Value
Tumor size	18.7±11.7	22.2±15.3	0.081
<20 mm, n (%)	91 (59.9)	108 (51.2)	-
≥20 mm, n (%)	61 (40.1)	103 (48.8)	-
Location			<0.001*
Upper third, n (%)	11 (7.2)	39 (18.5)	-
Middle third, n (%)	40 (26.3)	82 (38.9)	-
Lower third, n (%)	101 (66.4)	90 (42.6)	-
Location in the cross-sectional circumference			0.929
Lesser curvature, n (%)	67 (44.1)	94 (44.5)	-
Non-lesser curvature, n (%)	85 (55.9)	117 (55.4)	-
Ulceration (endoscopic), n (%)	2 (1.3)	7 (3.3)	0.226
Invasion depth			0.017*
Mucosa, n (%)	142 (93.4)	179 (84.8)	-
Submucosa, n (%)	10 (6.6)	32 (15.2)	-
Ulceration (histologic), n (%)	3 (2.0)	9 (4.3)	0.228
Histology			0.0971
Adenoma, n (%)	30 (19.7)	28 (13.3)	-
Adenocarcinoma, n (%)	122 (80.3)	183 (86.7)	-
Difficult ESD, n (%)	93 (61.2)	107 (50.7)	0.048*
Procedure time	77.1±41.4	78.7±57.0	0.243
≤60 min, n (%)	63 (41.4)	107 (50.7)	-
>60 min, n (%)	89 (58.5)	104 (49.3)	-
Self-completion, n (%)	113 (87.5)	200 (94.8)	0.001*
Complete resection, n (%)	151 (99.3)	200 (94.8)	0.017*
En bloc resection, n (%)	152 (100.0)	211 (100.0)	-
Severe complication, n (%)	3 (2.0)	12 (5.7)	0.079
Intra-procedual perforation, n (%)	0 (0)	3 (1.4)	-
Delayed perforation, n (%)	0 (0)	0 (0)	-
Delayed bleeding, n (%)	2 (1.3)	8 (3.8)	-
Aspiration, n (%)	1 (0.7)	1 (0.5)	-

Factors related to difficulty

**Figure 3:**
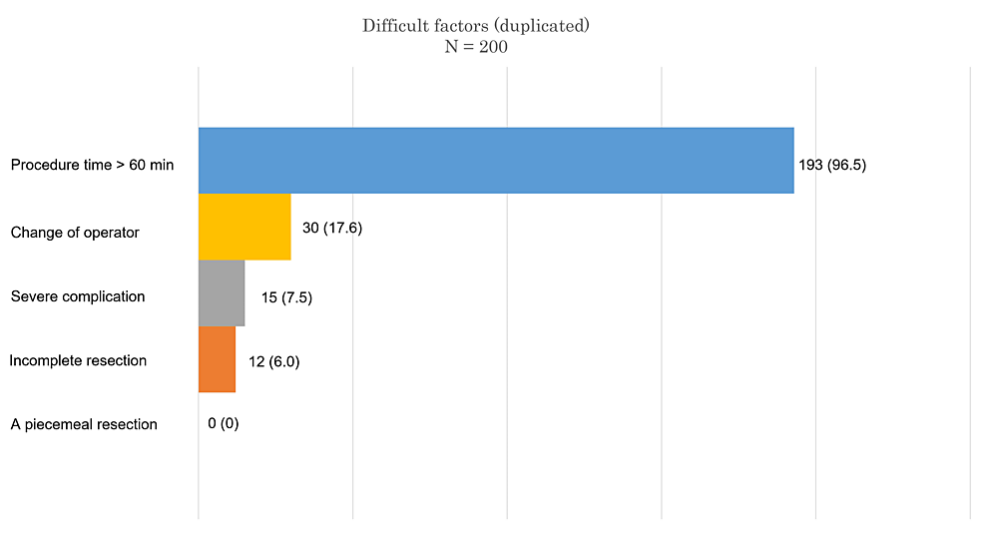
Details of difficult ESD items. ESD: endoscopic submucosal dissection

Among the reasons for ESD difficulty, the most common reason was procedure time >60 min (96.5%) (Figure 3). We analyzed the predictive factors associated with difficult ESD in both groups. Ulceration was excluded from ESD difficulty predicters in the analysis because of small number of lesions in both groups (the EB group: endoscopic, three cases; pathological, six cases; and the EI group: endoscopic, seven cases; pathological, nine cases).

In the EB group, the LC location (odds ratio {OR} 2.87, 95% confidence interval {CI} 1.433-5.758, p=0.002), cancerous histology (OR 2.49, 95% CI 1.105-5.616, p=0.044), and the non-lower third location (OR 2.44, 95% CI 1.164-5.132, p=0.017) were significantly associated with difficult ESD in univariate analyses. After adjustment by multivariate analysis, the LC location and cancerous histology were significantly related to difficult ESD.

In the EI group, lesion size larger than 20 mm (OR 4.84, 95% CI 2.705-8.676, p≤0.01), the LC location (OR 2.23, 95% CI 1.284-3.894, p=0.04), and submucosal invasion (OR 6.68, 95% CI 2.461-19.52, p<0.01) were significantly related to difficult ESD. In multivariate analysis, lesion size larger than 20 mm, the LC location, and submucosal invasion were significantly related to difficult ESD (Table 3).

**Table 3:** Comparison of the difficulty of ESD with clinicopathological findings of the lesions in each group. *Statistically significant. ESD: endoscopic submucosal dissection

Characteristics	Beginner group			Non-beginner group			Total		
	Difficult (n=93)	Not difficult (n=59)	p-Value	Difficult (n=107)	Not difficult (n=104)	p-Value	Difficult (n=200)	Not difficult (n=163)	p-Value
Tumor size	-	-	0.569	-	-	<0.001*	-	-	<0.001*
<20 mm, n (%)	54 (59.3)	37 (40.7)	-	35 (32.4)	73 (67.6)	-	89 (44.7)	110 (55.3)	-
≥20 mm, n (%)	39 (63.9)	22 (36.1)	-	72 (69.9)	31 (30.1)	-	111 (67.7)	53 (32.3)	-
Location	-	-	0.012*	-	-	0.064	-	-	0.018*
Non-lower third, n (%)	38 (74.5)	13 (25.5)	-	68 (56.2)	53 (43.8)	-	106 (61.6)	66 (38.4)	-
Lower third, n (%)	55 (54.5)	46 (45.5)	-	39 (43.3)	51 (56.7)	-	94 (49.2)	97 (50.8)	-
Location in the cross-sectional circumference, n (%)	-	-	0.002*	-	-	0.004*	-	-	<0.001*
Lesser curvature	50 (74.6)	17 (25.4)	-	58 (61.7)	36 (38.3)	-	108 (67.1)	53 (32.9)	-
Non-lesser curvature	43 (50.6)	42 (49.4)	-	49 (41.9)	68 (58.1)	-	92 (45.5)	110 (54.5)	-
Ulceration (endoscopic) positive n (%)	2 (100)	0 (0.0)	0.257	7 (100)	0 (0)	0.008*	9 (100)	0 (0)	<0.001*
Invasion depth n (%)	-	-	0.554	-	-	<0.001*	-	-	<0.001*
Mucosa	86 (60.6)	56 (39.4)	-	80 (44.7)	99 (55.3)	-	166 (51.7)	155 (48.3)	-
Submucosa	7 (70.0)	3 (30.0)	-	27 (84.4)	5 (15.6)	-	34 (80.9)	8 (19.0)	-
Ulceration (histologic) positive n (%)	3 (100)	0 (0)	0.163	7 (77.8)	2 (22.2)	0.097	10 (83.3)	2 (16.7)	0.045*
Histology n (%)	-	-	0.025*	-	-	0.745	-	-	0.255
Adenoma	13 (43.3)	17 (56.7)	-	15 (53.6)	13 (46.4)	-	28 (48.3)	30 (517)	-
Adenocarcinoma	80 (65.6)	42 (34.4)	-	92 (50.3)	91 (49.7)	-	172 (56.4)	133 (43.6)	-

## Discussion

In previous studies, factors related to difficult ESD performed by experts, such as larger size, the upper-third location, submucosal fibrosis, and submucosal invasive cancer, have been reported [14-16]. Yoon et al. reported that posterior wall lesions and upper-third lesions were associated with longer procedure time, incomplete resection, and perforation [17]. As described in these reports, the difficulty of ESD and the therapeutic effect differ depending on the lesion location and clinicopathological findings. Based on these factors related to ESD difficulty, beginners are advised to start performing ESD for lesions that do not meet these difficult conditions. Several reports have been published regarding the training of gastric ESD to beginners, and it is recommended that beginners start their experience with ESD operator from lesions located in the lower third of the stomach [8,22]. The reason for this recommendation for beginners is thought to be due to the characteristics of the lower third, such as fewer blood vessels in the submucosal layer and good lifting of the submucosa with injection. In the present study, the proportions of lesions in the non-lower third, with ulcers, and invading the submucosa were significantly higher in the EI group than in the EB group. Although the difference was not significant, larger lesions and cancerous lesions tended to occur more frequently in the EI group than in the EB group. This indicates that even at our hospital, beginners were prepared with cases without conditions that may make the ESD difficult.

**Figure 4:**
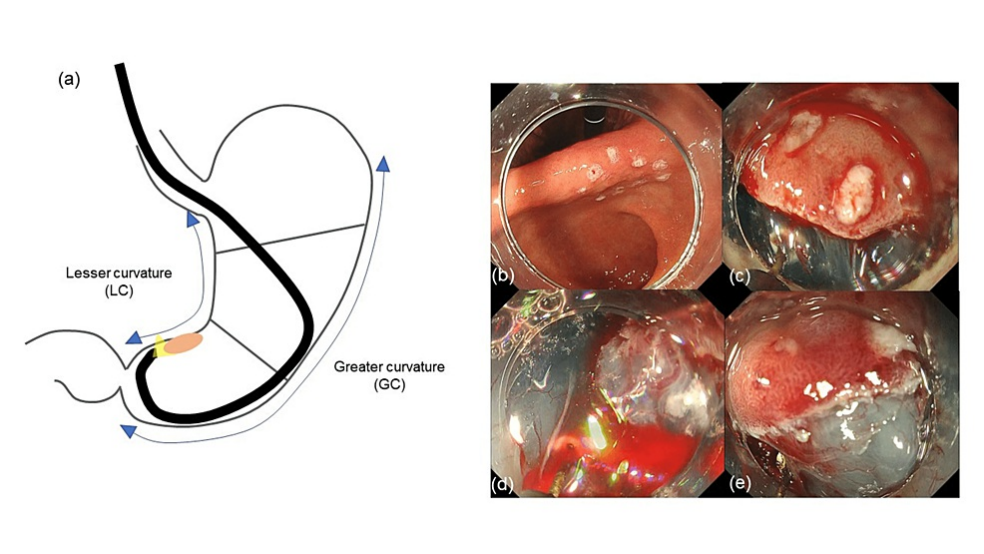
Schema of ESD-difficult case in the lesser curvature of the lower third (a). Intramucosal cancer located on the lesser curvature of the lower third (b). ESD with a retrograde position in a narrow space is required (c). The spilled blood hinders the clear visibility of the lesions when intraoperative bleeding occurs (d). Proximity of the muscle layer makes appropriate submucosal dissection difficult (e). ESD: endoscopic submucosal dissection

**Figure 5:**
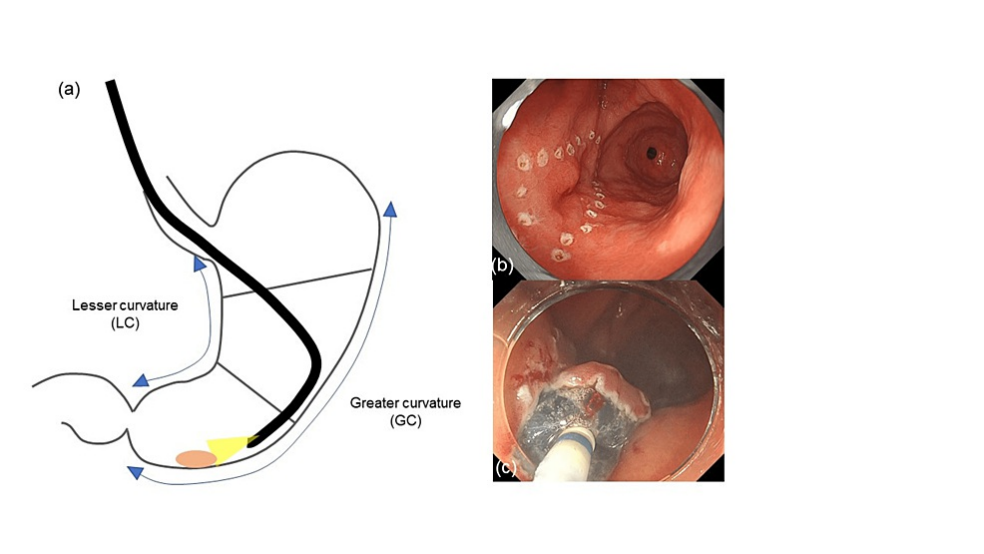
Schema of ESD-easy case of the lower third (a). Intramucosal cancer located on the anterior wall of the lower third (b). Appropriate distance from the lesion can be obtained, and the submucosal layer to be resected can be easily visualized (c). ESD: endoscopic submucosal dissection

In the present study, we found that the LC location in the cross-sectional circumference of the lesion is an independent factor for difficult ESD for not only beginners but also intermediates. This result differs from that reported by Yoon et al., which associated posterior wall lesions with longer procedure times and more frequent piecemeal and incomplete resections in ESD performed by an attending gastroenterologist. They considered that these ESD difficulties are partly explained by the difference in technical difficulty and poor visual field [14,17]. In our study, the results showed that the LC location had high ESD difficulty, and it was considered that the LC location might be a unique difficulty factor for inexperienced endoscopists. The causes of difficulty for beginners and intermediates performing ESD for lesions in the cross-sectional circumference in the LC for each location are as follows: in the lower third, the LC is in the direction of gravity, and when intraoperative bleeding occurs, the spilled blood gets collected, which hinders the clear visibility of the lesions. Moreover, usually, the ESD procedures for lesions located in the lower third are performed in the prograde position for lesions other than those in the LC portion (Figures 4a-4e); however, ESD with a retrograde position in a narrow space is required for lesions located in the LC. This situation may also make ESD difficult due to paradoxical movement of the scope (Figures 5A-5C). For lesions located in the middle third, it is often suitable to perform ESD in the retrograde position, especially for lesions in the LC. In this situation, it is often difficult to secure a visual field close to the lesion; to overcome this difficulty, it is necessary to take measures, such as adjusting the amount of air in the stomach, which may be difficult for non-experts. It was believed that the ESD-specific management of an endoscope, which is different from that of an observational endoscopy, increased the difficulty in treatment at this location.

The introduction of ESD operation to beginners under expert supervision is sufficiently effective and safe [7,20]. In this study, the complete resection rate in the EB group was 98.6% and a similar therapeutic effect was observed in the EI group (95.9%). Regarding complications, the perforation rate was 1.0%, and the delayed bleeding rate was 2.3% in the EB group, which was comparable to that in the EI group (1.2% and 2.8%, respectively).

This study suggested that although ESD introduction for beginners under expert supervision may be feasible; however, lesions located in the LC should be avoided as much as possible during case selection to complete ESD procedure in a reasonable amount of time.

Our study has several limitations. Firstly, this was a retrospective study conducted at a single center. For a more accurate verification, a multicenter prospective study is required. Secondly, beginner cases were already regulated and biased towards lesions that did not have known ESD difficulty factors, such as ulcer comorbid lesions. In fact, there were only three cases of ulcers in the EB group. However, it seems clinically inappropriate for beginners to perform ESD on lesions that are known to be difficult even for experts. Thirdly, this study did not analyze the learning curve in beginners. Only three of the six beginners completed 30 cases during the study period; therefore, it was not verified whether our method of introducing ESD to beginners was useful for learning ESD. Fourth, operator change was set as a difficult ESD condition in this study. The decision to change operator was made by the supervisors and it was possible that the decisions were not fully consistent among all supervisors. However, most of the difficult ESD cases took more than 60 min procedure time, and there was only one case where procedure time was less than 60 min even though the operator was changed. From this, it was considered that the difference in the judgment of changing operator by the supervisor did not greatly affect the results.

## Conclusions

In conclusion, lesions located in the LC should be avoided while introducing ESD to beginners. This lesion selection policy for ESD beginners should be considered to complete ESD procedure in a reasonable amount of time. Furthermore, further evaluation of the clinical significance of tumor location in the cross-sectional circumference in ESD performed by beginners is required to substantiate our recommendations.
